# Machine Learning Models for Predicting Freeze–Thaw Damage of Concrete Under Subzero Temperature Curing Conditions

**DOI:** 10.3390/ma18122856

**Published:** 2025-06-17

**Authors:** Yanhua Zhao, Bo Yang, Kai Zhang, Aojun Guo, Yonghui Yu, Li Chen

**Affiliations:** 1 Civil Engineering Department, Lanzhou Jiaotong University, Lanzhou 730070, China; zhaoyanhua1109@126.com (Y.Z.); yangbo4892@yeah.net (B.Y.); guoaojun@yeah.net (A.G.); yyh1056848662@163.com (Y.Y.); 2 Key Laboratory of Desert and Desertification, Northwest Institute of Eco-Environment and Resources, Chinese Academy of Sciences, Lanzhou 730030, China; 3 School of Railways and Architecture, Chongqing Vocational College of Public Transportation, Chongqing 402247, China; 15922851486@163.com

**Keywords:** low and subzero temperature, machine learning, SSA-ELM (Sparrow Search Algorithm-optimized Extreme Learning Machine), SHAP value (SHapley Additive exPlanations), empirical formulas

## Abstract

In high-elevation or high-latitude permafrost areas, persistent subzero temperatures significantly impact the freeze–thaw durability of concrete structures. Traditional methods for studying the frost resistance of concrete in permafrost regions do not provide a complete picture for predicting properties, and new approaches are needed using, for example, machine learning algorithms. This study utilizes four machine learning models—Support Vector Machine (SVM), extreme learning machine (ELM), long short-term memory (LSTM), and radial basis function neural network (RBFNN)—to predict freeze–thaw damage factors in concrete under low and subzero temperature conservation conditions. Building on the prediction results, the optimal model is refined to develop a new machine learning model: the Sparrow Search Algorithm-optimized Extreme Learning Machine (SSA-ELM). Furthermore, the SHapley Additive exPlanations (SHAP) value analysis method is employed to interpret this model, clarifying the relationship between factors affecting the freezing resistance of concrete and freeze–thaw damage factors. In conclusion, the empirical formula for concrete freeze–thaw damage is compared and validated against the prediction results from the SSA-ELM model. The study results indicate that the SSA-ELM model offers the most accurate predictions for concrete freeze–thaw resistance compared to the SVM, ELM, LSTM, and RBFNN models. SHAP value analysis quantitatively confirms that the number of freeze–thaw cycles is the most significant input parameter affecting the freeze–thaw damage coefficient of concrete. Comparative analysis shows that the accuracy of the SSA-ELMDE prediction set is improved by 15.46%, 9.19%, 21.79%, and 11.76%, respectively, compared with the prediction results of SVM, ELM, LSTM, and RBF. This parameter positively influences the prediction results for the freeze–thaw damage coefficient. Curing humidity has the least influence on the freeze–thaw damage factor of concrete. Comparing the prediction results with empirical formulas shows that the machine learning model provides more accurate predictions. This introduces a new approach for predicting the extent of freeze–thaw damage to concrete under low and subzero temperature conservation conditions.

## 1. Introduction

Concrete is the most extensively used material globally among the five major contemporary civil engineering resources: concrete, masonry, steel, wood, and synthetic materials [1,2]. The ongoing expansion of human living spaces and the variety of living environments have subjected many concrete structures to harsh conditions for extended periods. These conditions, such as freezing and thawing cycles [3], dry and wet cycles [4], and chemical erosion [5], significantly impact the durability of concrete. In cold regions, concrete buildings, bridges, and roads often suffer from varying degrees of freeze–thaw damage. This damage degrades the material properties of the concrete, posing significant risks to the safety of these structures [6,7,8].

**Figure 1:**
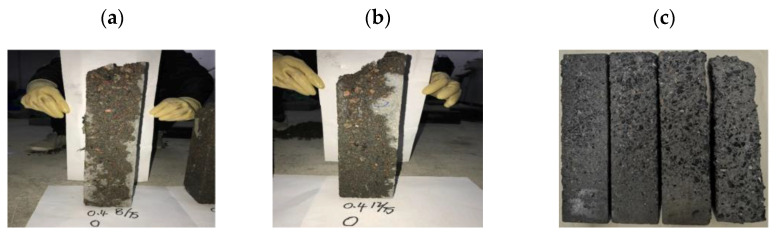
Concrete specimen damaged by freeze–thaw cycle [9,12].

For concrete structures constructed in high-elevation or high-latitude permafrost regions in northwest China, low and subzero temperature curing significantly affects the frost resistance of the concrete [9,10,11]. Figure 1 shows a concrete specimen damaged by freeze–thaw cycles. Currently, some scholars are studying the changes in concrete frost resistance under low and subzero temperature conditions. Dai et al. [9] investigated the frost resistance of the concrete conserved at −3 °C and found that curing at subzero temperatures reduced its frost resistance. Their results indicate that while concrete conserved at −3 °C can achieve the same strength as 28 d standard conserved concrete, it does not possess the same frost durability or service life. Jin et al. [12] studied how curing temperature affects the freeze–thaw resistance of hydraulic concrete. They discovered that after 200 freeze–thaw cycles, limestone powder concrete conserved for 28 d at 5 °C had the loosest structure and the widest cracks, followed by concrete conserved at 20 and 50 °C. Chen et al. [13] researched the relationship between conservation conditions (temperature and humidity) and the frost resistance of the concrete. They used nuclear magnetic resonance techniques to examine the microstructural damage of concrete after freeze–thaw cycles under various water-to-cement ratios and conservation conditions. Their findings revealed that as curing temperature and humidity decreased, the pore distribution and pore quality in the concrete increased. This insufficient curing dramatically impacted the frost resistance of concrete. Andisheh Zahedi et al. [14] investigated freeze–thaw (FT) deterioration in concrete specimens through an integrated assessment combining mechanical testing and microscopic analysis. They specifically applied pressure tensile (PT) and compressive strength tests, along with the Damage Rating Index (DRI). The findings demonstrate that both PT and DRI are effective and reliable for evaluating the microstructural condition of concrete exposed to freeze–thaw cycles. PT is especially useful for detecting early-stage damage, while DRI can identify key deterioration features across different stages of degradation. By assessing the durability factor and powder porosity before and after freeze–thaw (F-T) exposure, Sothyrak et al. [15] demonstrated that the drilled powder method—based on threshold powder porosity—is an effective approach for evaluating the resistance of concrete to F-T damage. Yang et al. [16] investigated the combined effects of chloride ions and F-T cycles on recycled concrete using the rapid freeze–thaw method. Their results showed that with increasing cycle numbers and chloride ion ingress, the formation of Friedel’s salt and gypsum accelerates, ultimately resulting in structural deterioration. Wang et al. [17] conducted a comprehensive investigation into the freeze–thaw damage and shear performance of “concrete-rock” composite structures by integrating nuclear magnetic resonance, scanning electron microscopy, and a self-developed high-precision saturated shear test device. Their study revealed that the interfacial transition zone between the concrete and rock was especially susceptible to freeze–thaw cycles, making it the primary region of damage within the composite structure. Li et al. [18] examined the deterioration mechanisms of wind-deposited sand concrete under freeze–thaw and carbonation-coupled freeze–thaw conditions. Their study showed that wind-deposited sand improves concrete durability by modifying its porosity and pore structure. In contrast, carbonation exacerbates freeze–thaw damage, mainly due to the formation of expansive products that cause surface cracking, thereby facilitating water penetration.

However, studies predicting freeze–thaw damage of concrete under low and subzero temperature curing are lacking. Most existing research focuses primarily on predicting freeze–thaw damage under standard conservation conditions. A freeze–thaw damage prediction model has been developed, primarily based on research into the factors affecting the frost resistance of the concrete. Rong et al. [19] developed a versatile freeze–thaw damage model that accounts for the uneven temperature distribution within concrete structures. They validated the model’s accuracy and reliability through a series of design tests. Bai et al. [20] developed macroscopic and microscopic freeze–thaw damage equations for wind-sand concrete. This was accomplished by introducing fatigue damage theory, based on the principles of irreversible thermodynamics and continuum damage mechanics. Jin et al. [21] performed freeze–thaw cycle and pore structure tests on concrete, developing a microscopic freeze–thaw damage model using fractal dimension as the independent variable. They analyzed the relationship between durability factors and the model parameters.

To advance the understanding of concrete durability under complex environmental conditions, Yan et al. [22] developed a coupled computational model to analyze the degradation of offshore structures subjected to both freeze–thaw cycles and sulfate erosion. This approach uses changes in porosity as an intermediate variable and was applied to simulate the deterioration of T-beams and columns in marine environments. Li et al. [23] introduced a freeze–thaw durability assessment method based on the fractal box-counting dimension of internal pores, which serves as a quantitative damage indicator. Expanding on experimental findings, Wang et al. [24] proposed a predictive model for freeze–thaw damage in polypropylene fiber-reinforced recycled concrete. The model incorporates different recycled aggregate replacement rates and polypropylene fiber contents to enhance its applicability across various material compositions. Li et al. [25] developed a strength degradation model for freeze–thaw foam concrete using the least squares method, achieving an error margin below 3.5% between theoretical predictions and experimental data. Zhang et al. [26] evaluated the early-age durability of concrete through the entropy weight method and proposed a GM-GA-BP neural network model to predict durability. Their model demonstrated strong accuracy, with an R^2^ value of 0.9822.

Traditional empirical freeze–thaw damage models have significant limitations in predicting concrete’s frost resistance. These models rely on empirical formulas derived under specific test conditions, making them inadequate for capturing the complex and variable environmental factors and material property differences encountered in real-world applications. As modern concrete compositions become more complex—with the incorporation of mineral and chemical admixtures—and service conditions increasingly diverse, including variations in freeze–thaw cycles, temperature gradients, and humidity fluctuations, the accuracy and applicability of traditional empirical formulas have been further constrained [27]. Thus, there is an urgent need for advanced predictive methods and assessment frameworks based on multi-parameter coupled analysis. Developing models that accurately characterize the intricate interactions among environmental conditions, material properties, and degradation mechanisms is crucial for improving durability design and lifespan prediction of concrete structures in cold regions.

As computing power has increased, researchers have begun using advanced artificial intelligence algorithms to solve complex problems. In particular, machine learning algorithms are being employed to predict the performance of concrete [28,29]. Hou et al. [30] developed a model to predict the shear strength of concrete beams using a genetic algorithm-optimized Back Propagation neural network. This model accurately forecasts the shear strength of ultrahigh-pressure concrete beams, offering valuable guidance for their design. Tanhadoust et al. [31] investigated the mechanical properties of normal-weight aggregate concrete and lightweight aggregate concrete at elevated temperatures. They used a long short-term memory (LSTM) neural network to predict the stress–strain relationships of normal-weight aggregate concrete and lightweight aggregate concrete mixtures under these conditions. The findings show that the LSTM model can successfully forecast the compressive strength, modulus of elasticity, and destructive strain relationships of normal-weight aggregate concrete and lightweight aggregate concrete mixes at elevated temperatures. Nguyen et al. [32] conducted a comprehensive review of artificial intelligence methods. They meticulously described, analyzed, and discussed the applicability, accuracy, and computational requirements of several major algorithms. Hiew et al. [33] compiled a comprehensive database of 228 axially loaded ultrahigh-performance concrete columns to develop three deep feed-forward neural network models. These models predicted the ultimate stress, ultimate strain, and stress–strain behavior of confined ultrahigh-performance concrete. The results indicated a high level of accuracy in capturing various stress–strain curves and showed strong alignment with experimentally measured responses. Wan et al. [34] used a deep neural network to optimize the peak load and toughness of concrete. The outcome indicated that the optimized concrete beams improved by 0.17% in maximum peak load, 14.13% in toughness, and 3.45% in mixing objectives compared to the original data. Lyngdoh et al. [35] utilized different machine learning models to predict concrete strength and found that the Extreme Gradient Boosting model achieved the highest performance.

In summary, machine learning exhibits a robust capacity for predicting the mechanical properties of concrete and the variations in its stress–strain behavior. However, the freeze–thaw damage of concrete under low and subzero temperature conservation conditions has rarely been addressed. This paper utilizes four machine learning models—extreme learning machine (ELM), LSTM, SVM, and Radial Basis Function Neural Network (RBFNN)—to predict the freeze–thaw damage factor of concrete under low and subzero temperature conservation conditions. Based on the prediction results, we propose a new optimization model called the Sparrow Search Algorithm Optimized Extreme Learning Machine (SSA-ELM). This study also employs SHapley Additive exPlanations (SHAP) value analysis to evaluate the relationship between input parameters and output outcomes. Finally, a comparison is made between the prediction results of the new machine learning model and those of the empirical formula model. Through the coefficient of concrete freeze–thaw damage prediction, further verify the precision and generalization abilities of the machine learning model. Our study tackles the challenge of predicting the relative dynamic elastic modulus (RDEM) of concrete under complex environmental conditions, such as freeze–thaw cycles—an important factor rarely modeled with machine learning despite its key role in durability assessment. Existing RDEM datasets are often small and fragmented, which limits the accuracy of previous models. To address these issues, we systematically integrate experimental data from multiple sources, providing a more comprehensive and robust analysis than earlier studies that used data from single conditions.

Traditional optimization methods, such as grid search and genetic algorithms, are commonly used for hyperparameter tuning in ELM. However, they often incur high computational costs and suffer from unstable convergence, especially when applied to high-dimensional concrete durability datasets with nonlinear degradation patterns. To overcome these limitations, we propose the SSA-ELM model, which incorporates the SSA—a bio-inspired optimizer that adaptively balances exploration and exploitation—into the ELM framework. This hybrid model excels at predicting concrete durability by effectively handling coupled environmental factors, such as freeze–thaw cycles combined with chloride attack, providing a reliable tool for service-life estimation where conventional models often fall short due to complex nonlinearities. Predicting frost resistance in permafrost regions remains challenging because of the intricate interactions between material properties and environmental conditions. Our study addresses this challenge by applying the SSA-ELM model, which offers several key advantages. From a scientific perspective, machine learning models can drive advances in frost resistance research through multi-scale mechanism modeling, optimization of small sample datasets, and enhanced interpretability using SHAP values to identify critical parameters. From an engineering standpoint, this technology supports intelligent monitoring systems, hybrid material design, and digital twin-based operation and maintenance. Nonetheless, issues such as data heterogeneity and cross-domain model generalization must be resolved to fully bridge the gap between precise laboratory predictions and comprehensive engineering lifecycle management.

## 2. Test Data

### 2.1. Data Sources and Analysis

**Figure 2:**
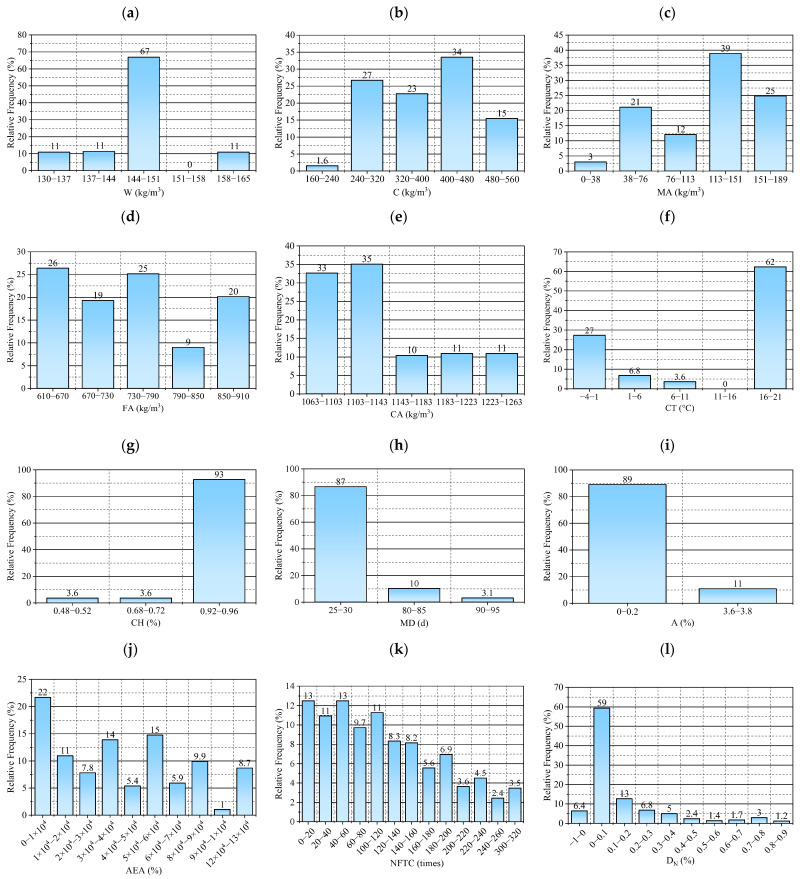
Distribution histograms of input and output parameters: (**a**) distribution interval of W dosage, (**b**) distribution interval of C dosage, (**c**) distribution interval of MA dosage, (**d**) distribution interval of FA dosage, (**e**) distribution interval of CA dosage, (**f**) distribution interval of CT, (**g**) distribution interval of CH curing humidity, (**h**) distribution interval of MD, (**i**) distribution interval of A dosage, (**j**) distribution interval of AEA dosage, (**k**) distribution interval of NFTC, and (**l**) distribution interval of *D_N_*.

In machine learning predictive modeling, a reliable database is essential for accurate predictions. The composition of the test data in the database must be internationally recognized. In addition, the database should meet the requirements of statistical indicators, and the data values in the database should uniformly cover all the value ranges that can be achieved by the characteristic values. Following these principles, this paper compiles data from the studies of Jin et al. [12], Dai et al. [9], Chen et al. [13], and Zhang et al. [36], resulting in a database of 576 samples. The data were extracted using WebPlotDigitizer (4.6-win32.x64 version), a software with key advantages. It features coordinate axis calibration tools to ensure precise data extraction. Additionally, it allows manual adjustment of data points, making it suitable for complex or low-resolution images. Table 1 presents the core parameter characteristics of the main datasets utilized in this study, offering a concise overview of the data foundation. As shown in Table 1., Jin et al. [12] selected three temperature conditions: 5 °C, 20 °C, and 50 °C. Dai et al. [9] and Zhang et al. [36] used two conditions: 20 °C and −3 °C, while Chen et al. [13] focused on three temperatures: 3 °C, 10 °C, and 20 °C. Since this study investigates the frost resistance of concrete under low negative temperature curing conditions, the 50 °C data from Jin et al.’s study [12] was excluded. The remaining data across all temperature conditions were included in the analysis. Including water–glue ratio maintenance, humidity maintenance days, air-entraining agent dosage, and so on. All these studies share the same independent variable parameters, which, along with the key dependent variable of relative dynamic elastic modulus, form the foundation of the database for this study. This database consists of 11 input parameters and 1 output parameter. Table 2 shows the statistical details of the dataset used for ML modeling. Figure 2 illustrates the histogram distribution of both input and output parameters.

As illustrated in Figure 2 and Table 2, W, C, MA, FA, CA, CT, CH, MD, AEA, A, and the NFTC are the input parameters selected. Because the dosage of the air-entraining agent significantly affects the frost resistance of the concrete, it is considered a separate variable and excluded from the general A category. As the output parameter, the freeze–thaw damage factor (*D_N_*) of concrete after freeze–thaw cycles was used. The specific ranges for the input parameters are as follows: W: 130–162 kg/m^3^; C: 180–536 kg/m^3^; MA: 0–180 kg/m^3^; FA: 624–908 kg/m^3^; CA: 1066–1261 kg/m^3^; CT: −3–−20 °C; CH: 0.5–0.95; MD: 28–90 d; AEA: 0–0.12%; A: 0.35–3.6%; NFTC: 0–300 cycles; *D_N_*: −0.03–0.87%. Concrete’s freezing resistance is governed by multiple factors. Among them, the water–cement ratio and air-entraining agents are particularly crucial, as they reduce capillary porosity and introduce uniformly distributed microbubbles that help relieve internal freezing pressure. Additional influential factors include the type of cement and the use of mineral admixtures, which contribute to the densification of hydration products; the properties of aggregates, which help reduce internal water retention; curing conditions, which ensure sufficient hydration; and chemical admixtures, which indirectly improve freeze resistance. Shafighfard et al. [37] selected 18 input features to predict the compressive strength of high-performance alkali-activated concrete using a machine learning model. In contrast, our study adopts a smaller set of parameters. This difference primarily stems from the difficulty in standardizing freeze–thaw data under low-temperature curing conditions, where variations in experimental design and data availability lead to inconsistent feature selection across studies. Therefore, we opted for a more concise and consistent feature set to enhance data comparability and model reliability. This reliable database ensures that the machine learning model can correctly forecast the *D_N_* of concrete. The formula for the *D_N_* is shown in Equation (1):

(1) $$D_{N}=1-\frac{E_{N}}{E_{0}}$$

where $E_{0}$ denotes the initial dynamic elastic modulus (%) of the concrete specimen and $E_{N}$ denotes the dynamic elastic modulus (%) of the concrete specimen after *N* freeze–thaw cycles.

### 2.2. Sensitivity Analysis of Characteristic Parameters

**Figure 3:**
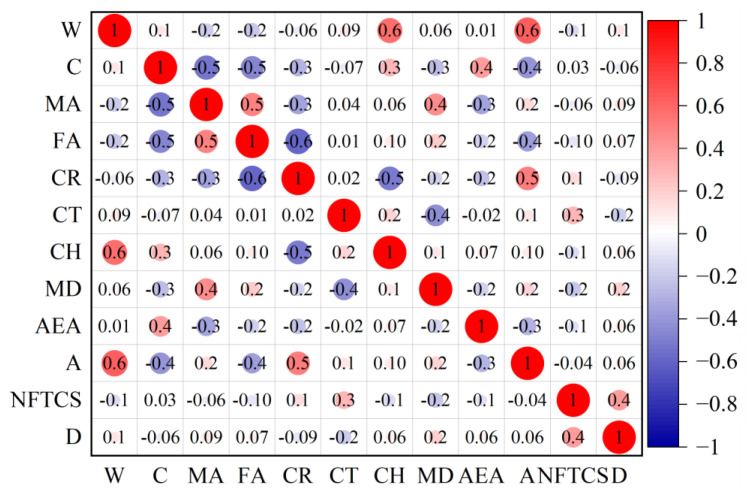
Correlation matrix plot between individual parameters.

If the plus and minus correlation coefficients among input parameters are excessively elevated, it suggests that individual factors are redundantly contributing to the output, complicating the assessment of their specific impacts. To gain a clearer understanding of the sensitivity between parameters, the Pearson correlation coefficient matrix is created by analyzing the database, as illustrated in Figure 3. The correlation coefficients between input parameters range from −0.6 to 0.6, indicating no significant correlation between them. Furthermore, the relevance of the association between input and output parameters is weak, ranging from −0.2 to 0.4. This suggests that all parameters are appropriate for constructing machine learning models.

**Figure 4:**
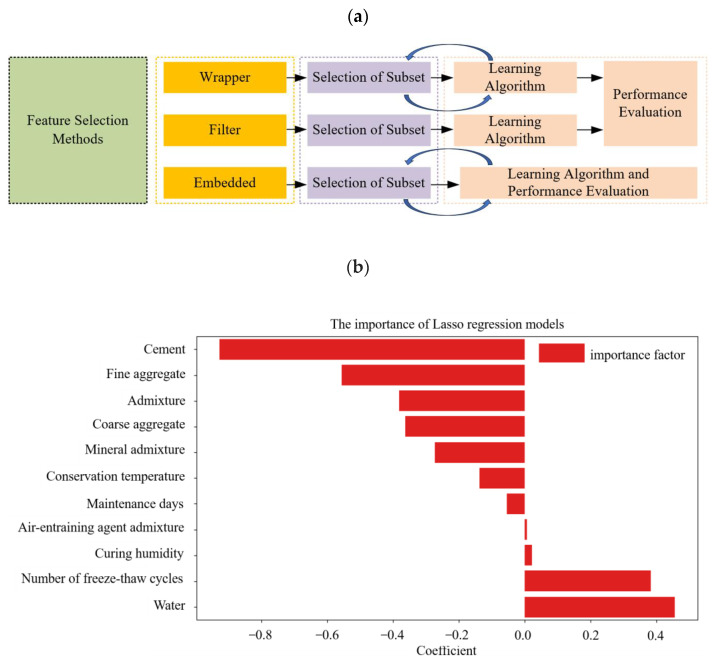
(**a**) Illustration of feature selection methods and (**b**) the importance of Lasso regression models.

Feature selection methods are crucial for identifying relevant features in datasets and are classified into three categories: wrapper, embedded, and filter techniques. Utilizing feature selection methods can significantly enhance the capabilities of machine learning models and provide them with tools to regulate predictive fluctuations. Figure 4a depicts the feature selection process adopted in this study. We employed the Least Absolute Shrinkage and Selection Operator (LASSO), which offers a significant advantage over traditional methods: it automatically selects relevant features by shrinking the regression coefficients of irrelevant or redundant variables to zero through L1 regularization. This approach is especially advantageous for datasets with numerous candidate features, among which only a few have a strong impact on the target variable. Figure 4 shows the feature importance distribution derived from the LASSO results. As illustrated, the freezing resistance of concrete is mainly governed by the water–cement ratio, air-entraining agents, and curing conditions—identified as the most influential factors. In comparison, mineral admixtures, aggregate grading, and chemical admixtures play secondary roles. Cement type and the number of freeze–thaw cycles exert indirect effects by modifying hydration processes and influencing damage levels. Notably, all selected features have nonzero importance coefficients. When considered along with Figure 4b, these results validate the inclusion of all selected variables in the development of the machine learning model.

## 3. Machine Learning Models

Given the complex correlations among the factors influencing freeze–thaw damage in concrete, this study utilized four machine learning models: SVM, ELM, LSTM, and RBFNN. Unlike the study by Shafighfard et al. [37], which investigates a broad spectrum of machine learning models, this study adopts a depth-oriented strategy by focusing on four model types, each with distinct algorithmic strengths. SVM: Selected for its ability to model nonlinear relationships among freeze–thaw parameters through kernel functions. ELM: Preferred for its high training speed, making it ideal for rapid evaluations across multiple scenarios. LSTM: Suited for capturing the time-dependent accumulation of damage over successive freeze–thaw cycles. RBFNN: Effective in detecting localized damage due to its sensitivity to spatial variations. These models were chosen for their specific advantages, which align with the characteristics of the dataset and the research goals. The principles and methodologies of these models have been detailed in previous studies [38,39,40], so this study offers only a brief overview. Building on the strong forecast performance of these four models, this study proposes an optimization model called SSA-ELM. This model retains the small sample size and fast computational prediction characteristics of the ELM while incorporating the robust optimization capabilities and rapid convergence speed of the SSA. Section 3.4 details the construction process of the SSA-ELM model. Table 3 provides the critical parameters of the four models. Based on empirical guidelines, the number of hidden layer nodes is set to 100 for ELM and 50 for SSA-ELM. This follows the general rule that the hidden layer size should be 1 to 10 times the number of input features (with an input feature dimension of 11), preventing dimensionality issues while ensuring sufficient model capacity. For LSTM, the initial learning rate is set to 0.02, selected to adapt to the gradient characteristics of the time series data. The learning rate decay factor is set to 0.8, with mild decay to balance convergence speed and parameter tuning accuracy. In RBFNN, feature scale differences are eliminated using Min-Max normalization, and the expansion rate is set to 100 based on grid search and simulation tests. Experiments show that this setting enables the Gaussian kernel function to effectively capture local features of the data distribution, and its generalization ability is confirmed after inverse normalization. Finally, for the SVM model, the penalty factor is set to 0.8 based on empirical guidelines, providing a moderate penalty for misclassified boundary samples while balancing model robustness and classification accuracy.

### 3.1. ELM

**Figure 5:**
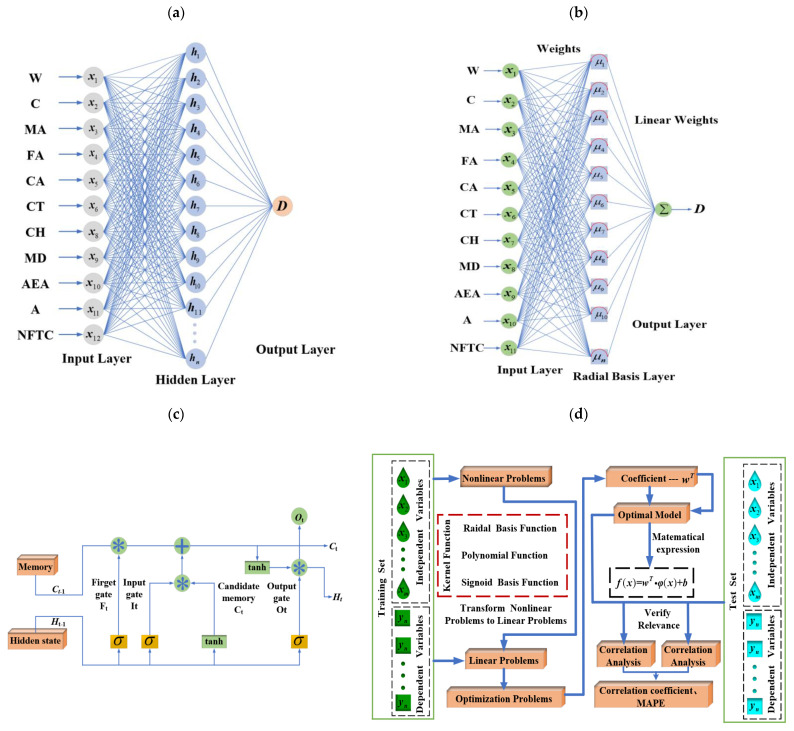
The analysis process of three machine learning models: (**a**) the analysis process of LSTM, (**b**) the analysis process of RBFNN, (**c**) the analysis process of the ELM algorithm, and (**d**) the analysis process of SVM.

ELM is an efficient, single-hidden-layer feedforward neural network. Its core feature is the random initialization of input weights and hidden-layer biases, followed by direct computation of output weights using the generalized inverse matrix, eliminating the need for iterative back-propagation. This approach offers key advantages, including fast training, high generalization capability, and deterministic global solutions [41]. Figure 5a shows the analysis process of the ELM algorithm. Structurally, ELM is made up of an input layer, a hidden layer, and an output layer. Suppose there are *N* samples $\left(x_{i}{,y}_{i}\right)$, where $x_{i}$ $={\left(x_{i1},x_{i2},\cdots ,x_{in}\right)}^{T}\in R^{n}$, $y_{i}={\left(y_{i1},y_{i2},\cdots ,y_{in}\right)}^{T}\in R^{m}$. The ELM model randomly generates weights and hidden layer deviations before training. For the ELM model with L hidden layer nodes and activation function f(x), the output feature can be formulated as Equation (2) [42].

(2) $$y_{t}={\sum_{i=1}^{L}\beta }_{i}f_{i}(w_{i}b_{i}x_{i})=h(x_{t})\beta$$

where *t* = 1, 2, …, *N*; $\beta_{i}$ is the threshold value of the neurons in the hidden layer; $\beta ={\left(\beta_{1}{,\beta }_{2},\cdots \beta_{L}\right)}^{T}$ is the connection rights matrix of the hidden layer and the output layer; $h(x_{t})$ is the mutation function from the input layer data to the eigenspace [42].

Since the randomly generated input weights ω and hidden layer biases *b* form different weight combinations W=(w, b, β), the training goal of ELM is to find the combination that minimizes the output error. Given inputs ω and *b*, the output matrix *H* [42] of the hidden layer is uniquely determined. The matrix of output weights is ${\beta =H}^{+}Y$, and $H^{+}$ is the Moore–Penrose generalized inverse of *H*. The matrix *H* is expressed in Equation (3).

(3) $$H=\left(\begin{array}{cccc} f({w_{1}}^{T}x_{1}+b_{1}) & f({w_{2}}^{T}x_{1}+b_{2}) & \cdots & f({w_{3}}^{T}x_{1}+b_{L})\\ f({w_{1}}^{T}x_{2}+b_{1}) & f({w_{2}}^{T}x_{2}+b_{2}) & \cdots & f({w_{3}}^{T}x_{1}+b_{L})\\ \vdots & \vdots & \ddots & \vdots \\ f({w_{1}}^{T}x_{N}+b_{1}) & f({w_{2}}^{T}x_{N}+b_{2}) & \cdots & f({w_{3}}^{T}x_{N}+b_{L}) \end{array}\right)$$

### 3.2. LSTM

LSTM is a specialized type of Recurrent Neural Network that addresses the gradient vanishing and explosion issues in traditional RNNs during long-sequence training. This is accomplished through the use of a gating mechanism. LSTM’s core structure consists of three control units: the forgetting gate, input gate, and output gate, which regulate information retention, updating, and output, respectively. In material science, LSTM is particularly effective for modeling temporal data, such as predicting the degradation of concrete performance during continuous freeze–thaw cycles. [43]. Figure 5b illustrates the analysis process of LSTM.

LSTM mitigates the problem of long-term perceptual loss caused by gradient vanishing during the training of traditional neural networks. Additionally, it aids in uncovering nonlinear relationships between characteristic parameters and the concrete *D_N_*. This capability makes LSTM suitable for developing a measurement model for freeze–thaw damage in concrete.

### 3.3. RBFNN

RBFNN is a three-layer feed-forward neural network recognized for its ability to approximate locally and its rapid convergence. The network consists of an input layer, a hidden layer with radial basis functions (RBFs) as activation functions, and an output layer. The core principle is to achieve function approximation through linear combinations of nonlinear radial basis functions. In material performance prediction, RBFNN models the complex nonlinear relationship between freeze–thaw damage factors and influencing variables. Its local response characteristics enable it to capture the non-uniform effects of key parameters on concrete durability with high precision. As illustrated in Figure 5c. The hidden layer simplifies the regression task by transforming input data into a high-dimensional space using radial basis function (RBF) [44]. The RBF is a mathematical function centered on a specific point, decreasing exponentially with increasing distance from the center. The Gaussian function, the most widely used RBF, serves as the activation function for the hidden layer.

The step size of weight updates during the training process is determined by the learning rate. It is a key parameter affecting the convergence and stability of training. A high learning rate results in significant weight updates in each iteration, potentially leading to not only faster convergence but also the risk of overshooting the optimal value. Conversely, a low learning rate results in smaller weight updates, which may slow convergence but lead to more stable and accurate updates [44].

### 3.4. SVM

SVM is a classic supervised learning algorithm that achieves effective classification by constructing an optimal hyperplane in the feature space. Guided by structural risk minimization, SVM finds the decision boundary that maximizes the margin between classes, minimizing training error and improving generalization. Compared to traditional machine learning methods, SVM has a strong mathematical foundation that ensures a globally optimal solution and avoids local minima. It offers key advantages: its decision-making relies on a limited set of critical data points (support vectors), enhancing computational efficiency, especially for large datasets. Additionally, SVM allows flexible balancing of model complexity and generalization through tuning the penalty parameter and kernel function. These benefits have led to its widespread use in research and applications, as shown in Figure 5d.

### 3.5. SSA-ELM

In machine learning and deep learning, the ELM is widely acclaimed for its impressive efficiency and superior generalization capabilities. Known for its remarkable learning speed, ELM can quickly adapt to new data and perform well on various tasks [42]. The generalization ability of ELM refers to its capacity to maintain stable performance across different datasets, leading to applications in fields such as image recognition and speech processing. However, ELM often faces inefficiencies when dealing with large datasets. This inefficiency primarily stems from the stochastic selection of initial weights and thresholds. Although this approach is simple and intuitive, it can limit performance because these parameters may not accurately reflect the data’s distributional characteristics. Consequently, the model may experience slow convergence and insufficient generalization during the learning process.

**Figure 6:**
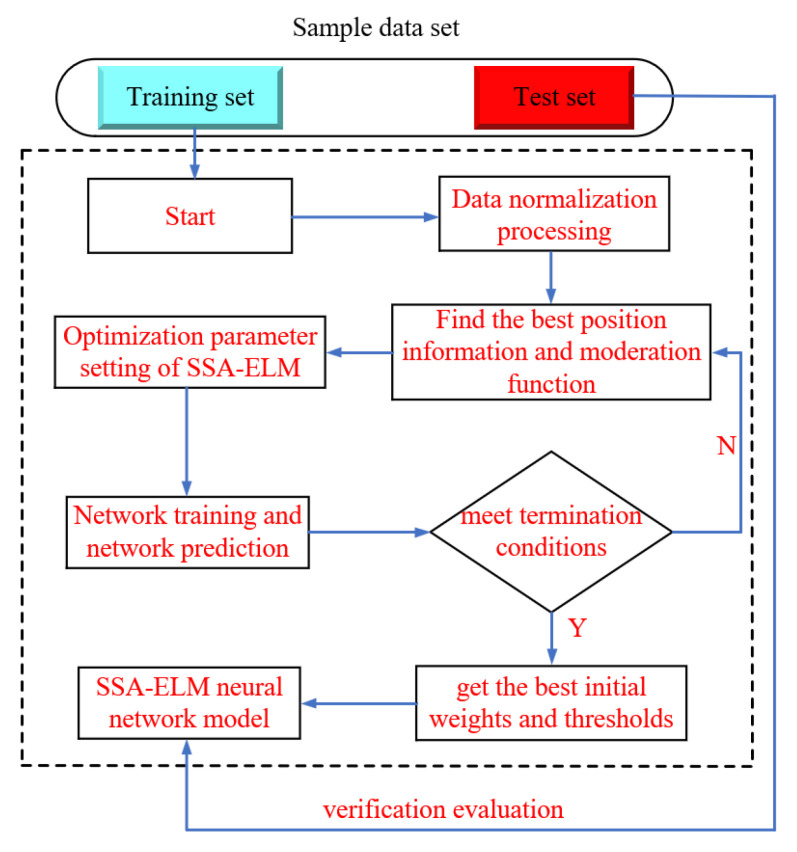
Analysis process of the SSA-ELM model.

To tackle this issue, this study introduces the SSA to enhance the initial parameter selection of the ELM [45,46]. The SSA is an optimization algorithm built on the behavior of communities in nature and optimizes the weights and thresholds of ELM effectively. The forecast accuracy and generalization ability of ELM are significantly improved by using SSA. This optimization method not only resolves the challenge of parameter setting in traditional ELM but also enhances its convergence speed and prediction accuracy. Moreover, the model improves both the global nature of parameter optimization and convergence speed while retaining the efficient characteristics of ELM’s single forward computation. Its unique group-based intelligent search strategy enhances adaptability and prediction accuracy in handling high-dimensional nonlinear data. As a result, the model is particularly well-suited for engineering prediction scenarios that require fast modeling and stable results. Figure 6 illustrates the analysis process of the SSA-ELM. The detailed steps of the SSA for optimizing ELM are as follows:

(1) Initialization: The sparrow population is incorporated into the model using the initialization algorithm. Parameters such as population size, learning rate, and the number of iterations are set.
(2) Random Sampling: An optimal sparrow individual is selected from the current population. Its position information is copied to the current position, and the fitness function for that position is calculated.
(3) Iterative Updating: Randomly select sparrow individuals from the population and modify their position information. Based on the fitness value and updated positions, eliminate the less optimal sparrow individuals and retain the better ones for the next round of competition. The SSA makes small adjustments to the parameters with each iteration, gradually bringing the estimated parameter value closer to its true value.
(4) Repeat Steps: Continue repeating Steps 2 and 3 until a predetermined termination condition is reached or the preset learning objective is achieved.
(5) Evaluation and Application: Evaluate the optimized ELM model using a test dataset to ensure its performance surpasses that of the original ELM.

## 4. Analytical Method

### 4.1. Data Pre-Processing

In machine learning, evaluation indicators often have varying scales and units, which can affect data analysis results. To eliminate these differences and ensure comparability between indicators, data standardization is necessary. By normalizing raw data, indicators are brought to the same order of magnitude, facilitating comprehensive and comparative evaluation. The most common method for this process is data normalization [47], as shown in Equation (4).

(4) $$Y=\frac{Z{Z-Z}_{\min}}{Z_{\max}{-Z}_{\min}}$$

where *Y* is the result of regularization, $Z_{\min}$ and $Z_{\max}$ are the min and max values for each parameter in the data sample, and *Z* is the sample value to be normalized.

### 4.2. Model Training and Testing Process

**Figure 7:**
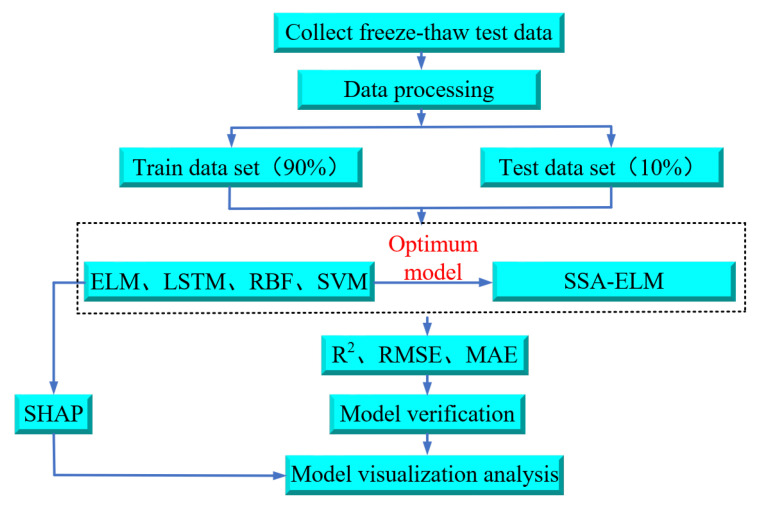
Flowchart of the research in this paper.

Figure 7 presents the research flowchart of this paper, which comprises five steps: data gathering, data handling, model training, optimization of the ELM model using the SSA, and model comparison, validation, and interpretation.

As illustrated in Figure 7, data were initially gathered from published studies on freeze–thaw tests of concrete under low and subzero temperature conservation conditions to train the machine learning model. A total of 576 samples are randomly split into two groups: 518 samples (90%) were used as a training dataset for the ELM, LSTM, SVM, and RBFNN models. After training, the remaining 58 samples (10%) were used as a test dataset to evaluate the performance and predictive capabilities of the trained models. The machine learning model is analyzed using evaluation metrics to identify the optimal ELM prediction model. Afterwards, the ELM model is optimized using the SSA. The prediction findings of the optimized SSA-ELM model are then compared with empirical formulas for verification. Finally, to gain deeper insights into the relationship between concrete *D_N_* and individual parameters, the model is interpreted using SHAP value analysis [48,49,50].

The SHAP value analysis technique quantifies the precise contribution of each feature using SHAP values. It ranks the significance of features and evaluates their effect on predicted values throughout the entire dataset. Additionally, it evaluates the effect of features on predicted values for individual samples. As an additive explanatory model, SHAP considers all features as “contributors”. For each forecast sample, the model generates a forecast value, and the SHAP value is assigned to each feature within that sample.

Suppose that the *i*-th sample is $x_{i}$ and the *j*-th feature of the *i*-th sample is $x_{\mathrm{ij}}$. The model’s predicted value for this sample is $y_{i}$, and the baseline for the entire model (usually the mean of the target variable for all samples) is $y_{\mathrm{base}}$. The total of the contribution values of all features constitutes the eventual forecast of the model, which can be indicated as Equation (5).

(5) $$y_{i}{=y}_{\mathrm{base}}+f(x_{i1})+f(x_{i2})+\cdots +f(x_{\mathrm{ik}})$$

where $f(x_{\mathrm{ij}})$ is the SHAP value for $x_{\mathrm{ij}}$. Intuitively, $f(x_{i1})$ is the contribution value of the first feature in the *i*-th sample to the final predicted value $y_{i}$. When $f(x_{i1})>0$, it means that the feature improves the accuracy of the predicted value and has a positive effect. Conversely, it means that the eigenvalue makes the forecasted value less accurate and has an inverse effect.

**Figure 8:**
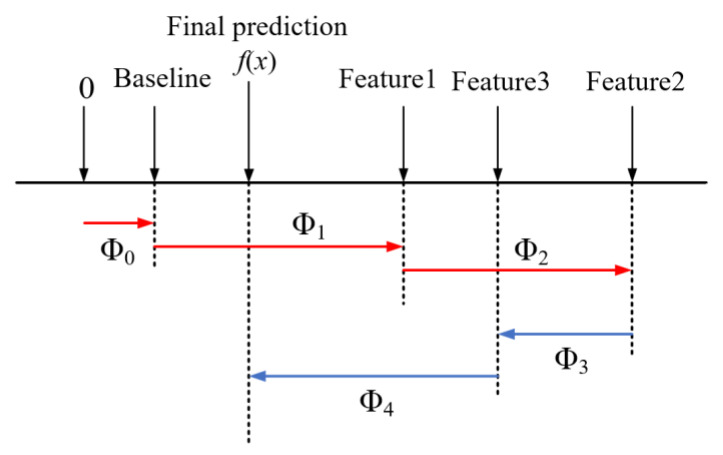
Schematic of SHAP model for four feature samples.

The primary advantage of SHAP values is their ability to reflect the influence of parameters in each sample and indicate whether their impact on the prediction results is positive or negative. For example, Figure 8 illustrates a schematic of four feature samples, with red representing positive contributions and blue indicating negative contributions. This approach effectively combines local and plenary explicability of machine learning models by showing the impact and contribution of each feature in the sample [47].

### 4.3. Evaluation Indicators

To assess the forecast accuracy of each machine learning model, this study employs three assessment indicators: the correlation coefficient (R^2^), root mean square error (RMSE), and mean absolute error (MAE) [51,52,53].

R^2^ is a crucial metric in regression analysis, indicating the square of the correlation between forecasted and actual outputs. Its value ranges from 0 to 1, with higher values denoting a stronger correlation between forecasted and target values. Specifically, an R^2^ value between 0.9 and 1 signifies a highly correlated model. RMSE measures the difference between forecasted and actual outputs in a machine learning model, while MAE assesses the average absolute error between them. Lower RMSE and MAE values, when compared to R^2^, indicate better behavior of the machine learning model. The definitions of R^2^, RMSE, MAE, and MAPE are provided in Equations (6)–(9). Compared to the indicators used by Shafighfard et al. [37], this study selects only four statistical indicators. Despite the smaller number, these indicators sufficiently support the core conclusions and offer precise, relevant insights. Similarly, Huang et al. [47] also used a limited set of indicators, which suggests that these four metrics are sufficient for evaluating the model.

(6) $$\mathrm{RMSE}=\sqrt{\frac{1}{n}\sum_{i=1}^{n}{\left(y_{i}-\hat{y_{i}}\right)}^{2}}$$

(7) $$\mathrm{MAE}=\frac{1}{n}\sum_{i=1}^{n}\left|y_{i}-\hat{y_{i}}\right|$$

(8) $$R^{2}=1-\frac{\sum_{i=1}^{n}{\left(y_{i}-\hat{y_{i}}\right)}^{2}}{{\sum_{i=1}^{n}\left(y_{i}-\bar{y_{i}}\right)}^{2}}$$

(9) $$MAPE=\frac{1}{n}\sum_{i=1}^{n}\left|\frac{y_{i}-\hat{y_{i}}}{y_{i}}\right|\times 100\%$$

where *n* is the number of test sets, $y_{i}$ is the true value, $\overset{\wedge }{y_{i}}$ is the corresponding forecasted value, and $\bar{y_{i}}$ is the mean value of $y_{i}$.

## 5. Results

### 5.1. Forecast Performance of the Four Models

**Figure 9:**
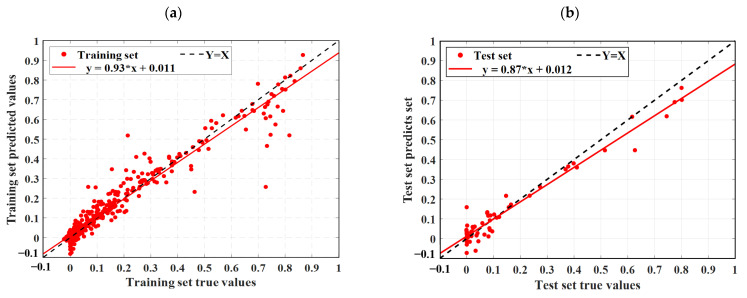

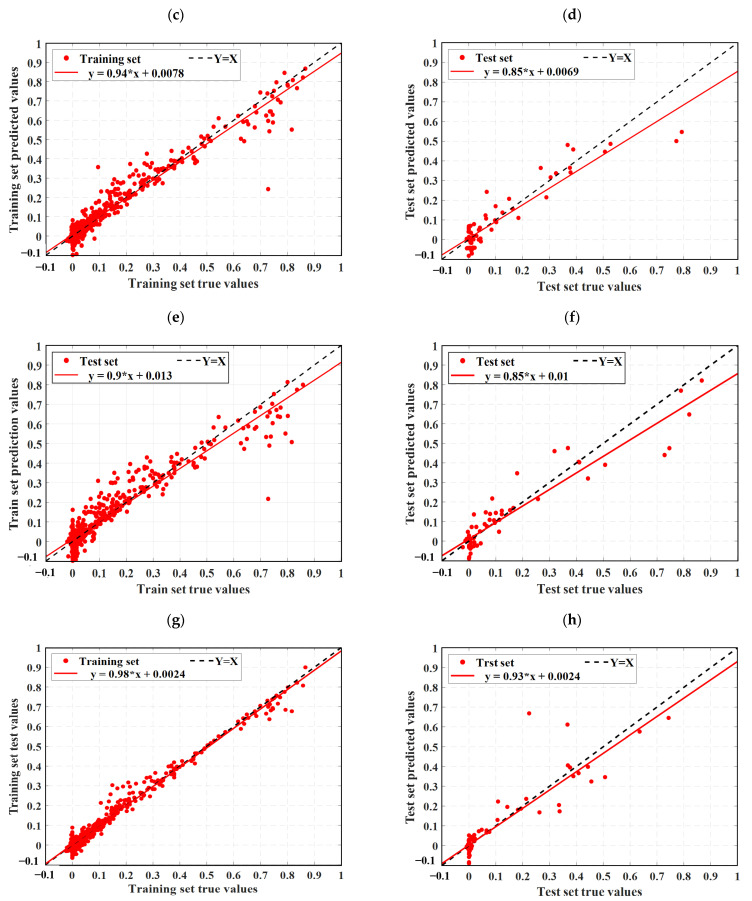

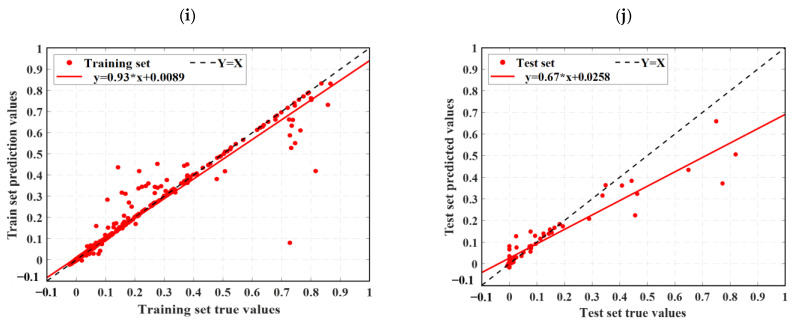
Scatter diagram of regression prediction results for four machine learning models: (**a**) LSTM training set predicted value results, (**b**) LSTM test set predicted value results, (**c**) ELM training set predicted value results, (**d**) ELM test set predicted value results, (**e**) RBF training set predicted value results, (**f**) RBF test set predicted value results, (**g**) SSA-ELM training set predicted value results, (**h**) SSA-ELM test set predicted value results, (**i**) SVM training set predicted value results, and (**j**) SVM test set predicted value results.

**Figure 10:**
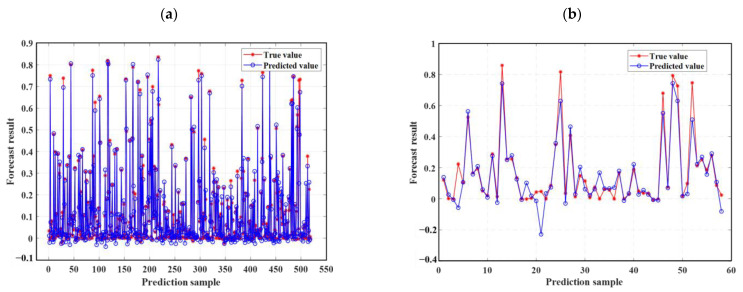
Comparison of prediction results and actual values of the SSA-ELM machine learning model on the training set and test set. (**a**) Comparison of training set with actual values; (**b**) Comparison of test set with actual values.

**Figure 11:**
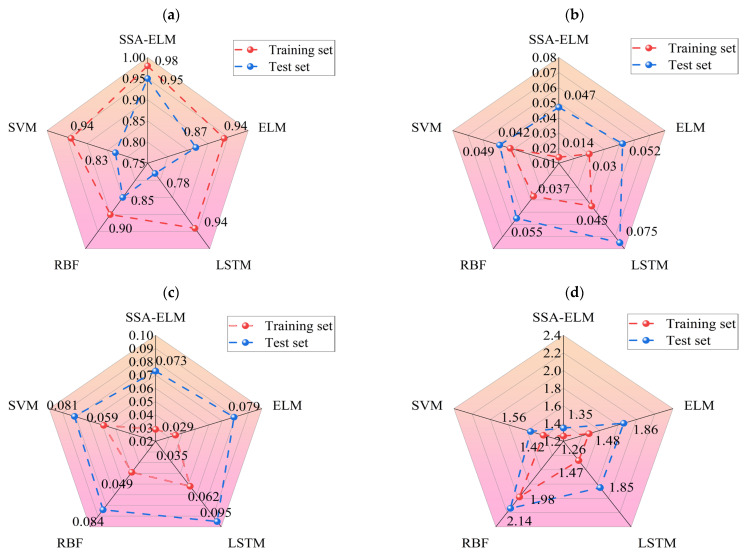
Evaluation metrics for four machine learning models: (**a**) R^2^ for the training and test sets of the four algorithms, (**b**) MAE for the training and test sets of the four algorithms, (**c**) RMSE for the training and test sets of the four algorithms, and (**d**) MAPE for the training and test sets of the four algorithms.

As described in Section 4.3, this study uses three statistical metrics to evaluate machine learning models. To facilitate an easy comparison of the forecast results, this section combines the outcomes of ELM, LSTM, RBFNN, SVM, and the optimization model SSA-ELM. Figure 9 shows the scatter diagram of the regression prediction results of the four machine learning models, illustrating the relationship between model predictions and test values. The horizontal axis shows the gathered concrete *D_N_*, while the vertical axis shows the predictions of the model. Samples nearer to the line Y = X indicate higher model accuracy, demonstrating that the model effectively catches the impact of input parameters on the concrete *D_N_*. This paper also includes a linear fit of the scatter distribution of the prediction results. The accuracy of these predictions is indicated by the slope of the fitted line. Figure 10 displays the prediction results of the SSA-ELM machine learning model for both the training and test sets, along with the real values. The predicted values align closely with the real values, with minimal error, demonstrating the model’s high prediction accuracy. Additionally, when compared to other machine learning models, SSA-ELM shows superior performance, further confirming its effectiveness and reliability in solving such problems. Furthermore, Figure 11 presents a radar chart comparing the evaluation metrics of the four machine learning models. Table 4 is a summary of the evaluation performance indicators.

Ideally, the data points in the graph should fall on a straight line along Y = X. As illustrated in Figure 9, the SSA-ELM is the best-performing machine learning prediction model. In this model, most points in both the training and test datasets fall on the line Y = X. Additionally, the slopes of the fitted straight lines for the training and test datasets in the SSA-ELM model are 0.98 and 0.93, respectively, making it the closest model to the ideal Y = X line. The SSA-ELM regression algorithm effectively captures the effect of input parameters on the *D_N_* of concrete, demonstrating strong forecast ability. As shown in Figure 11, the R^2^ values for the SSA-ELM prediction model are 0.98 for the training dataset and 0.95 for the test dataset. The results indicate that among the four models used in this paper, the SSA-ELM exhibits the best forecast and universalization ability for the *D_N_* of concrete. This superior performance is due to the SSA-ELM model’s strong adaptability to small datasets and its robust generalization capability.

**Figure 12:**
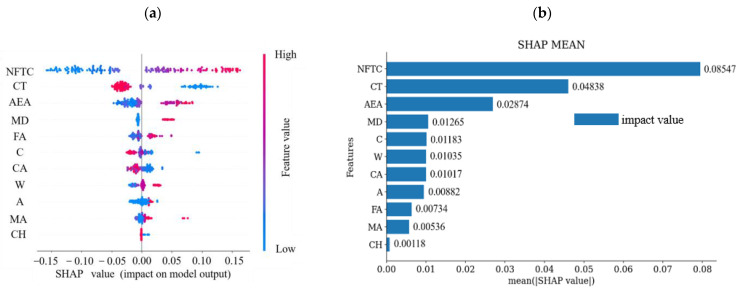
SHAP analysis: (**a**) distribution of SHAP values for 11 feature parameters and (**b**) feature significance analysis based on SHAP.

In this study, we used SHAP value analysis to interpret machine learning models. Figure 12 displays the distribution of SHAP values for each parameter, highlighting their influence patterns. The horizontal axis shows the SHAP values, showing the impact of parameters on the model output; the vertical axis then sorts the parameter contributions based on total SHAP values of all samples. Each point represents a sample, stacked vertically by sample size, with color indicating the eigenvalue. Figure 12a shows that the NFTC is the most critical parameter. The SHAP value increases as the NFTC increases, leading to a higher *D_N_* in concrete, which indicates accelerated damage. The results indicate that the NFTC positively affects the prediction of the *D_N_* of concrete. In many freeze–thaw tests, NFTC is the most intuitive influence. This result confirms that NFTC is not only the most direct influence but also one of the most critical factors. Additionally, higher conservation temperatures negatively affect the predicted results due to the presence of low and subzero temperatures. The effects of air-entraining agent dosage, admixture, and the number of days of maintenance follow the same trend as the NFTC.

Figure 12b illustrates the importance analysis plot of the feature parameters, derived by averaging the SHAP values of each characteristic in all the samples. It is evident from Figure 12b that the NFTC has the most dramatic influence on the *D_N_* of concrete. Conservation temperature follows, with a SHAP value that is 57% of that of NFTC. In contrast, conservation humidity has the least effect, with a SHAP value that is only 1.38% of that of NFTC.

**Figure 13:**
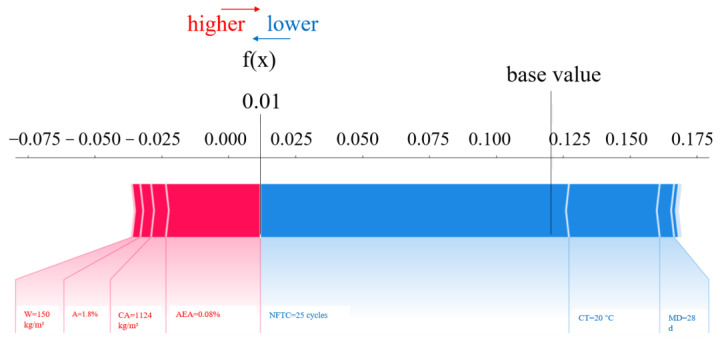
Results of SHAP interpretation for a single sample.

SHAP provides both global and local interpretations of the dataset. As depicted in Figure 13, SHAP breaks down the final forecast into the total of contributions from all input parameters. The red bars show parameters that positively affect the *D_N_* of concrete, while the blue bars represent parameters with a negative effect. The lengths of the color bars represent the corresponding addition or subtraction in values, quantifying the influence of each parameter. Huang et al. [47] applied the SHAP method to enhance the interpretability of machine learning models, addressing the common “black box” problem. Their findings quantitatively confirmed that corrosion rate has the greatest influence on bond strength. In contrast, this study focuses on the bonding behavior between steel and concrete, emphasizing different material characteristics. The study results indicated that air-entraining agents (0.08%) and admixtures (0.018%) positively impacted the *D_N_* prediction within a certain range. Conversely, the NFTC (25 cycles), curing temperature (20 °C), and maintenance days (28 d) negatively affected the prediction of the *D_N_* of concrete. Air-entraining agents reduce freeze–thaw damage by introducing uniformly distributed microscopic air bubbles into the concrete. These bubbles provide cushioning space during water freezing, alleviating internal stresses caused by volumetric expansion. Additionally, admixtures enhance freeze–thaw resistance by refining the pore structure (reducing harmful macropores), lowering the water-cement ratio, or increasing overall densification. Repeated freeze–thaw cycles expand internal microcracks and increase pore water pressure, ultimately causing spalling and strength loss. Moreover, low-temperature curing can delay cement hydration, leading to insufficient strength development, higher porosity, and diminished frost resistance. Insufficient curing time—such as shorter periods compared to longer ones—prevents concrete from achieving optimal compactness and full frost resistance. Poor curing conditions, including inadequate humidity, can also increase defects even after 28 days, further weakening the frost resistance of air-entrained concrete.

### 5.2. Comparison of Machine Learning Model and Empirical Formula Prediction Performance

To validate the suitability and effectiveness of hybrid machine learning models in forecasting concrete *D_N_*, we combined the analysis results from Section 5.1. The SSA-ELM demonstrated the best forecast performance and was used to compare its results with those calculated by empirical formulas from the literature.

Currently, empirical formulas are predominantly used to predict the freeze–thaw resistance of concrete. Although these methods have limitations, they remain reliable. For instance, Wang et al. [11] and Xia et al. [54] employed exponential functions to forecast the freeze–thaw damage of concrete, as illustrated in Equations (10) and (11).

Wang et al. [11]:

(10) $$D_{x}=\alpha_{1}e^{b_{1}n}+c_{1}$$

where $\alpha_{1}$, $b_{1}$, and $c_{1}$ are the fitting coefficients.

Xia et al. [41]:

(11) $$D(n)=1-\exp(-{\left(\frac{n}{\varepsilon }\right)}^{\beta })$$

where β is the Weibull shape factor, and ε is the scale factor; *n* is the NFTC.

**Figure 14:**
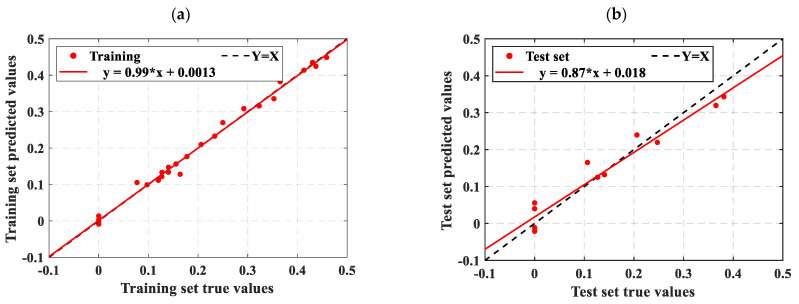
Prediction results of the SSA-ELM model on Wang et al. [11] data: (**a**) training set prediction results and (**b**) test set prediction results.

**Figure 15:**
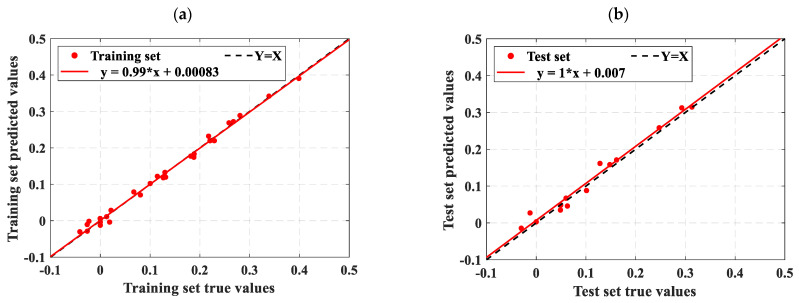
Prediction results of the SSA-ELM model for Xia et al. [54] data: (**a**) training set prediction results and (**b**) test set prediction results.

The freeze–thaw damage data from the studies by Wang et al. [11] and Xia et al. [54] were predicted using the SSA-ELM model, which demonstrated optimal forecast performance. The computational accuracy of both empirical and machine learning models is presented in Table 5. Figure 14 and Figure 15, respectively, illustrate the prediction results of the SSA-ELM model for the data from Wang et al. [11] and Xia et al. [54].

Figure 11 shows that most scatter points closely align with the ideal line Y = X, indicating minimal error between the predicted and experimental values. The slope of the fit line further confirms this small error. The R^2^ values for both the training and test datasets are higher than those reported by Wang, with the test set R^2^ value notably reaching 0.93. The SSA-ELM model predicts the *D_N_* of concrete more accurately than empirical formulas. This accuracy is due to machine learning models considering a wider range of influencing factors, whereas empirical formulas only account for a few intuitive ones. Figure 15 further supports this conclusion. As shown in Table 5, Xia et al. [54] found correlation coefficients greater than 0.94 when correlating the *D_N_* to the NFTC for various types of concrete. Additionally, the overall data analysis using the SSA-ELM model shows that the correlation coefficients for both the test and training datasets exceed 0.97. This demonstrates the feasibility of using the SSA-ELM model to predict the *D_N_* of concrete. The model optimizes the initial parameters of the ELM, including input layer weights and hidden layer settings, using the Sparrow Search Algorithm (SSA). This method overcomes the instability associated with random initialization in traditional ELMs, improving both prediction accuracy and generalization ability. The model also demonstrates enhanced nonlinear fitting capabilities, effectively capturing the multifactorial effects of freeze–thaw cycles and material proportions. Moreover, it is not restricted to the specific concrete types and freeze–thaw conditions studied here; it can be applied to concrete with varying mixing ratios, different freezing environments (e.g., minimum temperatures, freeze–thaw cycles, and cooling rates), and diverse geographical and climatic regions (e.g., cold areas, plateaus, and coasts). The model’s adaptability and generalization ability enable it to manage the complex nonlinear relationships between concrete material properties, environmental factors, and freeze–thaw damage. This makes it a reliable tool for assessing concrete freeze durability in various engineering contexts. Similarly, Huang et al. [47] compared the prediction results of machine learning models with those from empirical formulas. They found that machine learning models yield more accurate and user-friendly predictions, consistent with the findings of this study. Although the specific models and research focus differ, both studies highlight the promising potential of machine learning applications in this field.

### 5.3. Feature Parameter Selection Based on Machine Learning Model Analysis

In Section 2.2, this paper employs the Pearson correlation coefficient method for input feature selection. However, to achieve better predictability with fewer feature parameters, this section combines the analysis results from Section 5.1 with the feature selection method detailed in Section 2.2 to re-select more suitable input features.

To achieve better forecast performance with fewer characterization parameters, this study categorized the parameters into three groups: raw material parameters, environmental parameters, and material parameters. The raw material parameters include water, cement, mineral admixtures, fine aggregates, and coarse aggregates. Environmental parameters include maintenance temperature, maintenance humidity, number of maintenance days, and NFTC. Material parameters include air-entraining agents and admixtures. As shown in Figure 11, fine aggregate and cement exhibit the highest correlation with the *D_N_* among the raw material parameters. Fine aggregates increase the compactness, compressive strength, and durability of concrete. Cement, being the primary cementitious material, is crucial in engineering practice. Among the environmental parameters, the number of free NFTC ze-thaw cycles, conservation temperature, and conservation days significantly impact the *D_N_* of concrete. The *D_N_* rises with the NFTC. Conversely, the frost resistance of the concrete improves with higher conservation temperatures and longer conservation periods. Among the material parameters, air-entraining agent dosage has the most dramatic influence on the *D_N_* of concrete. The freeze resistance of concrete first increases and then decreases with the amount of air-entraining agent dosage. This indicates that an ideal quantity of air-entraining agent dosage can improve the freeze resistance of concrete.

**Figure 16:**
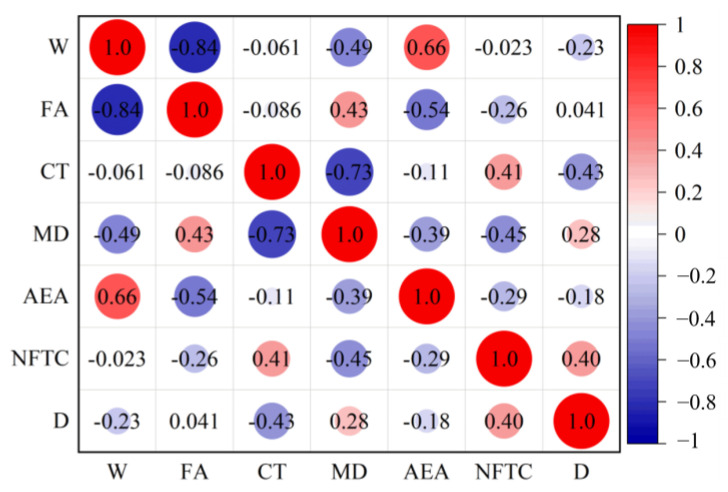
Correlation matrix plot between the 6 characteristic parameters.

Based on the above considerations, the final input characterization parameters selected are cement, fine aggregate, conservation temperature, curing days, NFTC, and air-entraining agent. Figure 16 presents the correlation matrix for each feature parameter after reselecting the parameters. As shown, the correlation coefficients for each input parameter range from −0.84 to 0.66. Only cement and fine aggregate have a negative correlation coefficient of −0.84. The negative correlation coefficient between cement and fine aggregate is high because both are raw materials for concrete, resulting in a double contribution to the predicted results. However, based on the analysis in Section 5.1, this study decided to use both cement and fine aggregate as input characterization parameters. Additionally, the correlations between the input and output parameters are weak, ranging from −0.43 to 0.4. This indicates that all six reselected feature parameters can be used to construct machine learning models. The concrete *D_N_* are predicted using the four models adopted in this study, and the predictions are shown in Table 6.

Table 6 shows that the forecast ability of the four machine learning models improves overall after re-selecting the feature parameters, except for SSA-ELM, which has a decrease in R^2^. Notably, ELM, LSTM, SVM, and RBFNN exhibit significant improvements, with R^2^ values exceeding 0.90 for both the training and test datasets. The analysis shows that reducing the feature parameters enhances the prediction performance of three models. Notably, ELM achieved the best results, with R^2^ values of 0.97 for the training dataset and 0.92 for the test dataset after the parameter reduction. Conversely, only SSA-ELM showed a decrease in R^2^ for both datasets, with the test set R^2^ dropping to 0.89. Not all four machine learning prediction models achieve optimal performance with fewer feature parameters. For datasets with limited features, the stochastic nature of SSA-ELM proves less effective, resulting in poorer predictions. Therefore, SSA-ELM is more suitable for scenarios with multiple input parameters, leading to more accurate predictions. ELM, LSTM, SVM, and RBFNN demonstrate significant improvement in prediction performance after reducing feature parameters, primarily due to their strong generalization abilities. Notably, ELM excels with its faster learning speed and high-precision prediction capability, making it the top performer post-feature reduction. The feature reduction process, or dimensionality reduction, may remove weakly correlated but collectively important features that the SSA-ELM model relies on to capture nonlinear patterns, resulting in valuable information loss. While this process reduces overfitting by simplifying the input space, it can also oversimplify complex high-dimensional interactions, limiting SSA-ELM’s ability to fully leverage these relationships.

## 6. Conclusions

This study aims to propose a new machine learning model to predict the *D_N_* of concrete under low and subzero conservation conditions. SHAP value analysis is used to determine the effect of each input variable on the *D_N_*. Additionally, this paper compares the prediction performance of empirical formulas and machine learning models for concrete freeze–thaw damage. The main findings are as follows:

(1) After evaluating different machine learning models, it is found that the SSA-ELM forecast model outperformed the ELM, LSTM, SVM, and RBFNN models. The SSA-ELM model demonstrated excellent performance in predicting *D_N_* of concrete under low and subzero temperature conservation conditions. Additionally, reducing the feature parameters improved the prediction accuracy of ELM, LSTM, SVM, and RBFNN due to their strong generalization abilities, with correlation coefficients all greater than 0.90. Although the correlation coefficient of the SSA-ELM test dataset slightly dropped to 0.89, it still shows good forecast performance.
(2) A method using SHAP values was introduced to analyze the effect of each input variable on the *D_N_* of concrete. SHAP value analysis offers both a global interpretation of the entire dataset and a local interpretation of individual data points. Globally, the NFTC emerges as the most significant parameter, positively influencing the prediction of the *D_N_*. However, locally, when the number of freeze–thaw cycles reached 25, it negatively affected the prediction of the *D_N_*.
(3) Comparison of the SSA-ELM model’s prediction results with empirical formulas reveals that the machine learning model demonstrates high accuracy. In contrast, empirical formulas, constrained by a limited number of variables, show relatively poor forecast performance.
(4) The SSA-ELM model bridges the gap between theoretical durability predictions and practical engineering applications. It provides rapid and accurate durability number forecasts under multifactorial exposures, enabling various practical uses. For instance, it aids preventive design by optimizing mix proportions to achieve target service life through sensitivity analysis. It also supports condition-based maintenance by utilizing real-time sensor data—such as temperature and moisture—to initiate interventions before the RDEM drops below safety thresholds. This approach minimizes unnecessary over-design, including excessive use of AEA, while preventing premature failures. However, the model’s accuracy relies heavily on the quality and breadth of its training data, and its reliability diminishes with small sample sizes or under extreme conditions like ultralow temperatures.

## Figures and Tables

**Table 1:** The parametric characteristics of the data.

Data Sources	Water-Cement Ratio	CT	CH	MD	AEA
Jin et al. [12]	0.45	50 °C, 20 °C, 5 °C	95%	28, 90	0.01%
Dai et al. [9]	0.28, 0.32, 0.36, 0.40	20 °C, −3 °C	95%	28	0.00%, 0.05%, 0.08%, 0.12%
Chen et al. [13]	0.35, 0.40, 0.40	20 °C, 10 °C, 3 °C	95%, 75%, 50%	28	0.03%
Zhang et al. [36]	0.24, 0.31, 0.38	−3 °C, 20 °C	95%	28, 84	0.00%, 0.03%, 0.06%, 0.09%

**Table 2:** Statistical details of the dataset used for ML models.

	W	C	MA	FA	CA	CT	CH	MD	AEA	A	NFTC
Mean	147.36	382.29	61.23	746.93	1135.69	12.28	0.92	35.67	0.04	0.41	110.42
Std	8.23	95.79	68.29	86.09	59.4	10.29	0.095	19.57	0.04	1.12	84.4
Min	130	180	0	624	1066	−3	0.5	28	0	0.35	0
25%	144	317	0	657	1081	−3	0.95	28	0.0001	0.013	50
50%	150	375	38	741	1124	20	0.95	28	0.0003	0.019	100
75%	150	469	118	813	1154	20	0.95	28	0.0006	0.028	175
Max	162	536	180	908	1261	20	0.95	90	0.12	3.6	300

**Table 3:** Critical parameters of the four models.

Model	Correlation Parameter	
	Parameter Name	Corresponding Value
ELM	Activation function	Sig
	Number of hidden layer nodes	100
LSTM	Initial learning rate	0.02
	Learning rate decay factor	0.8
RBFNN	Expansion rate of the RBF	100
SVM	Penalty factor	0.8
SSA-ELM	Population size	5
	Number of hidden layer neurons	50

**Table 4:** Summary of performance evaluation indicators.

Algorithm	Test-Set/Training-Set (%)	Best Performance			
		R^2^	MAE	RMSE	MAPE
ELM	1/9	0.94	0.052	0.079	1.86
LSTM	1/9	0.94	0.075	0.095	1.85
RBF	1/9	0.90	0.055	0.084	2.14
SVM	1/9	0.94	0.049	0.081	1.56
SSA-ELM	1/9	0.98	0.047	0.073	1.35

**Table 5:** Comparison of computational accuracy between empirical formulas and machine learning models.

Data Sources	R^2^ in the Original Article	R^2^ of the SSA-ELM Model	
		Test Set	Training Set
Wang et al. [11]	0.83	0.93	0.99
Xia et al. [54]	All greater than 0.9	0.97	0.99

**Table 6:** Comparison of computational precision of four machine learning models.

Model	Evaluation Index							
	R^2^		MAE		RMSE		MAPE	
	Train Set	Test Set	Train Set	Test Set	Train Set	Test Set	Train Set	Test Set
ELM	0.97	0.92	0.0159	0.0479	0.0354	0.0686	1.235	1.387
LSTM	0.91	0.91	0.0333	0.0468	0.0613	0.0517	1.494	1.827
RBFNN	0.95	0.92	0.0247	0.0323	0.0459	0.0593	1.381	1.529
SVM	0.92	0.90	0.0354	0.0375	0.0654	0.0576	1.374	1.961
SSA-ELM	0.97	0.89	0.0568	0.0812	0.0537	0.0334	1.249	1.994

## Data Availability

The original contributions presented in this study are included in the article. Further inquiries can be directed to the corresponding author.

## Abbreviations

W	water
MA	mineral admixture
CA	coarse aggregate
CH	curing humidity
AEA	air-entraining agent
NFTC	number of freeze–thaw cycles
LSTM	Long Short-Term Memory
SVM	Support Vector Machine
C	cement
FA	fine aggregate
CT	conservation temperature
MD	maintenance days
A	admixture
ELM	Extreme Learning Machine
RBFNN	Radial Basis Neural Networks
SSA-ELM	Sparrow Algorithm Optimized Extreme Learning Machine
